# Embryonic motor activity and implications for regulating motoneuron axonal pathfinding in zebrafish

**DOI:** 10.1111/j.1460-9568.2008.06418.x

**Published:** 2008-09

**Authors:** Evdokia Menelaou, Erin E Husbands, Robin G Pollet, Christopher A Coutts, Declan W Ali, Kurt R Svoboda

**Affiliations:** 1 Department of Biological Sciences, Louisiana State UniversityBaton Rouge, LA 70803, USA; 2 Department of Biological Sciences, University of AlbertaEdmonton, AB, Canada; 3 Centre for Neuroscience, University of AlbertaEdmonton, AB, Canada

**Keywords:** axon, motoneuron, Rohon–Beard, sensory neurons, zebrafish

## Abstract

Zebrafish embryos exhibit spontaneous contractions of the musculature as early as 18–19 h post fertilization (hpf) when removed from their protective chorion. These movements are likely initiated by early embryonic central nervous system activity. We have made the observation that *narrowminded* mutant embryos (hereafter, *nrd*^*−/−*^) lack normal embryonic motor output upon dechorionation. However, these mutants can swim and respond to tactile stimulation by larval stages of development. *nrd*^−/−^ embryos exhibit defects in neural crest development, slow muscle development and also lack spinal mechanosensory neurons known as Rohon–Beard (RB) neurons. At early developmental stages (i.e. 21–22 hpf) and while still in their chorions, *nrd* siblings (*nrd*^*+/?*^) exhibited contractions of the musculature at a rate similar to wild-type embryos. Anatomical analysis indicated that RB neurons were present in the motile embryos, but absent in the non-motile embryos, indicating that the non-motile embryos were *nrd*^−/−^ embryos. Further anatomical analysis of *nrd*^−/−^ embryos revealed errors in motoneuron axonal pathfinding that persisted into the larval stage of development. These errors were reversed when *nrd*^−/−^ embryos were raised in high [K^+^] beginning at 21 hpf, indicating that the abnormal axonal phenotypes may be related to a lack of depolarizing activity early in development. When activity was blocked with tricaine in wild-type embryos, motoneuron phenotypes were similar to the motoneuron phenotypes in *nrd*^−/−^ embryos. These results implicate early embryonic activity in conjunction with other factors as necessary for normal motoneuron development.

## Introduction

Neural activity is a key factor in regulating neural development. In many systems, neuronal activity guides developmental processes such as synapse formation and neurotransmitter expression (Shatz, 1990; Katz & Shatz, 1996; Borodinsky *etal.*, 2004). Classic evidence indicating that communication via action potentials is essential for normal synaptic formation arises from studies of the visual system. When Stryker & Harris (1986) infused tetrodotoxin into eyes of kittens several weeks postnatally, during a critical developmental period, neural activity in the retinal ganglion cells was blocked. Ultimately, this reduced excitatory input to the lateral geniculate nucleus (LGN) neurons and prevented the appropriate segregation of the LGN afferents as they projected to their targets.

Research using zebrafish and *Drosophila* illustrates a role for neural activity in normal central nervous system development. In the zebrafish *macho* (*mao*^−/−^) mutant, retinal ganglion cells have reduced Na^+^ currents and thus cannot fire action potentials (Gnuegge *et al.*, 2001). Consequently, the retinal ganglion cell projections in *mao*^−/−^ larvae are de-stabilized at their targets by 6 days post fertilization. Motoneurons of mutant flies having deficient Na^+^ current (the *nap* mutant) exhibit an increase in synaptic connections, likely via sprouting. The *nap* fly phenotype can be phenocopied by infusing drosophila larvae with tetrodotoxin (Keshishian *et al.*, 1994; Jarecki & Keshishian, 1995).

Spinal neurons are spontaneously active while their axons are projecting to their post-synaptic targets (Spitzer *et al.*, 2004). This activity can also impact nervous system development. Increasing the frequency of spontaneously occurring calcium transients in *Xenopus* neurons can alter transmitter phenotype (Borodinsky *et al.*, 2004). In developing chicks, the frequency of spontaneously occurring activity can influence motoneuron axonal pathfinding (Hanson & Landmesser, 2004, 2006).

In developing zebrafish, spontaneously occurring motor output occurs between ∼18 and 27 h post fertilization (hpf). In very young embryos (19.5 hpf), this output is likely caused by primary motoneurons releasing acetylcholine onto post-synaptic muscle pioneers and the muscle fibers adjacent to the muscle pioneers (Melancon *et al.*, 1997).

We have been studying the zebrafish mutant known as *narrowminded. nrd*^−/−^ embryos have disrupted neural crest development and lack spinal mechanosensory neurons known as Rohon–Beard (RB) neurons (Artinger *et al.*, 1999). They also lack muscle pioneers and slow (red) muscle because the adaxial cells transfate into multinucleated fast muscle fibers (Roy *et al.*, 2001). We show that *nrd*^−/−^ embryos also lack the embryonic muscle twitches early in development. We then characterize motoneuron axonal morphology in *nrd*^−/−^ embryos. Motoneuron axonal anatomy was altered in a manner similar to neurons from other systems where neural activity has been reduced, but did not resemble the anatomy of motoneuron axons in embryos where muscle pioneers have been ablated (Melancon *et al.*, 1997). When compared with motoneurons from *you-too* mutants (*yot*), which lack adaxial cells (van Eeden *et al.*, 1996; Zeller *et al.*, 2002), the motoneuron phenotypes in *nrd*^−/−^ mutants were much less severe. Pharmacological manipulation with the sodium channel blocker tricaine phenocopied the motoneuron phenotypes observed in *nrd*^−/−^ embryos while providing a depolarizing drive during early embryonic development with KCl restored the abnormal motoneuron morphology.

## Methods

### Zebrafish embryos and maintenance

Fertilized eggs were obtained from natural mating of adult zebrafish (*narrowminded* and several wild-type lines) according to the *Zebrafish Book* (Westerfield, 1995). Adult fish were maintained at 28.2°C with a 14/10-h light–dark schedule. Embryos were collected from identified heterozygous carriers for the *nrd* mutation or from wild-type adults within 3 h of spawning, rinsed and raised in 10-cm Petri dishes containing embryo medium. The *narrowminded* line (*nrd*^*m805*^) was kindly provided by Dr Kristin Artinger at the University of Colorado Health Sciences Center. Animal protocols were approved by the Institutional Animal Care and Use Committee at Louisiana State University and by the Canadian Council for Animal Care (for experiments performed at the University of Alberta).

### Behavior

Embryonic motor behavior was analyzed between 19 and 27 hpf with the aid of video-rate microscopy. Behavior of embryos in the chorion and out of the chorion was monitored with the aid of a Kohu video camera mounted to a Zeiss Stemi 2000C microscope and simultaneously captured to VCR tape. At the lowest magnification, the behavior (bends of the spinal musculature) of 16–20 embryos could easily be resolved. The behaviors were taped in 5- to 10-min epochs and the tapes were subsequently analyzed. The number of bends/twitches of the spinal musculature for each individual embryo was counted minute by minute for each epoch of recorded activity. Behavioral experiments were performed at room temperature (∼26 °C).

### Motoneuron and RB neuron anatomy

Whole-mount immunohistochemistry was carried out using a modified version of our previously published protocol (Svoboda *et al.*, 2001, 2002; Pineda *et al.*, 2006). Briefly, embryos or larvae were anesthetized in 0.1% MS222 (tricaine), fixed in 4% paraformaldehyde overnight at 2–4 °C, and then stored in PBST (PBS containing 0.1% Tween 20). On day1 of the protocol they were permeabilized and then incubated in a primary antibody overnight at 2–4°C. Primary antibodies were used as listed in Table 1 and all dilutions were prepared in PBST [anti-acetylated tubulin (aat), 1 : 1000; zn1, 1 : 200; znp1, 1 : 250; zn5, 1 : 1000; zn12, 1 : 250]. The antibodies zn12 and aat were used to label RB neurons. Zn1 was used to label primary motoneuron somata and znp1 was used to label primary motoneuron axons. The antibody zn5 was used to label secondary motoneuron axons. It is no longer in production, but zn8, which is a duplicate isolate of the same hybridoma as zn5 (Kawahara *et al.*, 2002), is available from the Developmental Studies Hybridoma Bank at The University of Iowa, Iowa City, Iowa, and the Zebrafish International Research Center at the University of Oregon (see Table 1).

**Table 1:** Information pertaining to primary antibodies

Antibody	Source	Concentration	Epitope	References
Anti-acetylated tubulin	SIGMA	1 : 1000	Acetylated tubulin	Svoboda *et al.* (2001)
Anti-engrailed (4D9)	DSHB*	1 : 200	Engrailed, muscle pioneers	Patel *et al.* (1989), Ekker *et al.* (1992)
zn1	DSHB*	1 : 200	Neuronal marker (cytoplasmic)	Trevarrow *et al.* (1990)
znp1	DSHB*	1 : 250	Motoneuron axons	Trevarrow *et al.* (1990)
zn5	DSHB*,†	1 : 1000	DM-GRASP, secondarymotoneuron axons	Fashena & Westerfield (1999)
zn12	DSHB*	1 : 250	HNK-1, neuronal axons	Metcalfe *et al.* (1990)
F59	DSHB*	1 : 50	Myosin heavy chain, adaxial(slow) muscle cells	Crow & Stockdale (1986),Miller *et al.* (1989)
F310	DSHB*	1 : 250	Myosin light chain, fastmuscle cells	Crow & Stockdale (1986)

* Developmental Studies Hybridoma Bank.

† Available as zn8 from the Developmental Studies Hybridoma Bank and the Zebrafish International Resource Center.

The following day, the samples were washed for 60 min and then incubated in a fluorescent secondary anti-mouse antibody conjugated to Alexa 546 or Alexa 488 (1 : 1000 dilution in PBST; Molecular Probes, Eugene, OR, USA) for 90 min to reveal primary antibody labeling. They were then rinsed in PBST for another 60 min and prepared for image analysis.

Single focal plane images of the fluorescent signals were acquired with an ORCA-ER digital camera (Hamamatsu) mounted to a Zeiss Axiovert 200M inverted microscope utilizing a 20×, 40× objective and either a green fluorescent protein (GFP) or rhodamine filter cube. This Zeiss microscope is equipped with an ApoTome. In some instances, images were acquired using a Leica confocal microscope (excitation laser line 543 nm, 40× oil objective). All of the acquired images of motoneuron axons were digitally processed with the aid of Adobe Photoshop 7.0 (Adobe Systems, San Jose, CA, USA). Using the invert function, the GFP (Alexa 488) signal or rhodamine (Alexa 546) signals were converted to a black-and-white inverted image and all analyses were then performed on these inverted images. We directed our efforts to the mid-region of the spinal cord found above the yolk sac extension. This region was reliably imaged after the embryos were mounted under coverslips. Quantification of axonal anatomical data was performed as described in our previously published work (Svoboda *et al.*, 2002). A minimum of five embryos or larvae were analyzed across developmental stages or experimental condition. Values are presented as the percentage of segments analyzed per embryo that had normal ventral axons, the percentage of dorsal segments analyzed per embryo that were innervated, or the percentage of ventral primary axons analyzed per embryo that exhibited normal morphology.

In some experiments, z stacks were acquired through the region of spinal cord overlying the yolk sac extension. These images were acquired with a 40× oil objective. The stacks were then projected and rotated using IMARIS 5.7 (Bitplane Inc., St Paul, MN, USA). This was done to confirm the results obtained with the single focal plane imaging utilizing the 20× objective.

### Muscle anatomy

The monoclonal antibodies F59 (1 : 50 dilution; Crow & Stockdale, 1986; Developmental Studies Hybridoma Bank, University of Iowa), F310 (1 : 250; Crow & Stockdale, 1986; Developmental Studies Hybridoma Bank, University of Iowa) and 4D9 (1 : 200 dilution; Patel *et al.*, 1989; Developmental Studies Hybridoma Bank, University of Iowa) were used to label slow muscle fibers (F59), fast muscle fibers (F310) and to detect engrailed (4D9), a marker of muscle pioneers in zebrafish. Monoclonal antibody labeling was detected with a fluorescent secondary anti-mouse antibody conjugated to Alexa 546 or Alexa 488 (1 : 1000 dilution in PBST; Molecular Probes).

We also used *Draq* 5 (Biostatus Limited, Shepshed, UK) to label muscle nuclei. Although it is a vital dye used to label nuclei in living cells, we found that it also labels nuclei in fixed tissue. Larvae were first processed through our immunohistochemistry protocol prior to *Draq 5* labeling. *Draq 5* (1 μL/mL PBST) was then added directly to an embryo lying on a slide and subsequently cover-slipped. After 30 min, a Leica confocal microscope (excitation laser line 633 nm, 40× oil objective) was utilized to image the *Draq 5* signal.

### Muscle histology

Larvae were fixed overnight at 2–4°C in 4% paraformaldehyde, and then rinsed overnight in PBS with 0.1% Tween 20. The PBST was removed and completely replaced with distilled water. A dehydration series beginning with 30% ethanol was followed in which over the course of 2 h, ethanol was gradually added to the water. The solution was then entirely replaced with 95% ethanol and then with 100% ethanol. The embryos were then infiltrated for a total of 5 h in 30, 50, 75 and 100% LR White Resin (Electron Microscopy Sciences). The 100% infiltration step was performed twice and then the samples were embedded. Cross-sections of 500 nm were cut from the region above the center of the yolk sac extension with a DuPont 5000 ultramicrotome, mounted on glass slides, stained with toluidine blue (in 2% sodium borate) and imaged with the aid of a digital camera (RT SPOT; Roper Scientific, Duluth, GA, USA) mounted to a Nikon upright microscope.

### Electrophysiology

Miniature endplate currents (mEPCs) were recorded from the fast muscle fibers of wild-type, *nrd*^*+/?*^ and *nrd*^−/−^ larvae at approximately 72 hpf. Fish were anesthetized in 0.01% tricaine, pinned through the notochord to Sylgard-lined glass slides, and perfused with extracellular saline containing (in mm) 134 NaCl, 2.9 KCl, 2.1 CaCl, 1.2 MgCl_2_, 10 HEPES, 10 glucose and 0.001 tetrodotoxin (Tocris, Avonmouth, UK). The nerve cord at the junction of the spinal cord and the hindbrain was pinched and severed with a fine pair of forceps. A section of the skin overlying the trunk was removed to allow access to axial muscles. As slow fibers in *nrd*^−/−^ larvae do not exist, we only compared the miniature endplate current (mEPCs) recorded from fast muscle fibers. Slow fibers from wild-type and heterozygous siblings were easily identified (Todd *et al.*, 2004; Coutts *et al.*, 2006) and were gently removed with a pipette immediately prior to recording. Whole-cell patch-clamp recordings were performed on axial fast fibers in voltage clamp mode (Hamill *et al.*, 1981). The pipette solution contained (in mm) 130 CsCl, 2 NaCl, 10 HEPES, 10 EGA, 2 CaCl_2_, 4 MgATP and 0.4 LiGTP. Polished pipettes were pulled from thin-walled glass (World Precision Instruments, Sarasota FL, USA) and had tip resistances of 1.0–1.8 MΩ resulting in whole cell series resistance values of 1.6–3.6 MΩ. Series resistances were monitored carefully and the recordings were abandoned if they changed by 15% or greater. All series resistances were compensated by 90% and fibers were voltage clamped at −60 mV throughout the recordings. Recordings were performed with an Axopatch 200B and mEPCs were captured with pClamp 8.1 software. All mEPCs were analyzed offline with Axograph X software. Data were sampled at 50 KHz and low-pass filtered at 10 kHz.

### Tricaine exposure

Tricaine (MS222) was purchased from Sigma and made as per Westerfield (1995). It was made fresh daily for each experiment and diluted to final concentrations in embryo medium.

### Statistics

All behavioral, anatomical and electrophysiological values are reported as means ± SEM. Student’s *t*-tests were performed to test for significance, which was assigned if the *P*-value was < 0.05. SigmaStat software was used for statistical analyses and data were plotted in SigmaPlot or Origin. Single and double exponential fits of the mEPC decay component were determined by comparing the sum of squared errors (SSEs) of the fits (Clements & Westbrook, 1991; Legendre, 1998). A double exponential fit was assigned when the SSE was significantly less than that for a single exponential fit (SSE 1 exp > SSE 2 exp; Student’s *t*-test; *P*< 0.05).

## Results

### Embryonic motor behavior of the *narrowminded* mutants

**Fig. 1:**
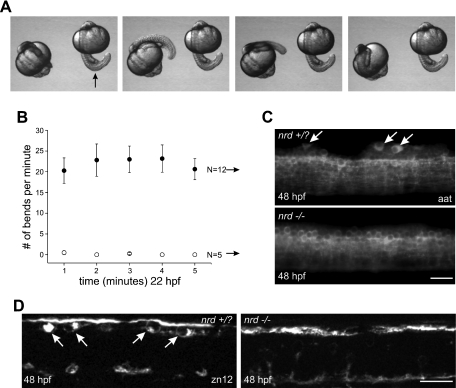
*Narrowminded* mutants do not exhibit an increase in musculature twitch rates upon dechorionation. (A) Two 22-hpf embryos that were dechorionated are shown in the field of view. In these experiments, embryos were observed and videotaped for 30 min. We noticed in our initial experiments that some embryos lacked movements when dechorionated (embryo denoted by black arrow). (B) Behavioral examination quantifying the number of bends that occurred for each embryo for each minute. The embryos that lacked movement were separated from the rest of the group and along with the motile embryos, processed for immunohistochemistry. (C and D) The motile embryos possessed aat- or zn12-positive RB neurons arrows at 48 hpf. Those that did not exhibit any movements lacked RB neurons. Scale bars, 20 μm.

Zebrafish embryos display bends of the musculature when removed from their chorions as early as 18 hpf. The frequency of these contractions peaks at about 19–20 hpf and then declines gradually (Saint-Amant & Drapeau, 1998). When we first began studying *narrowminded*, we dechorionated embryos between 23 and 26 hpf and noticed that a fraction of the embryos did not move upon dechorionation. We wondered if the non-motile embryos were in fact *nrd*^−/−^ embryos. To determine this, we combined behavioral and anatomical analyses of embryos spawned from heterozygote carriers of the *narrowminded* mutation. Embryos that did not exhibit spontaneous contractions of the musculature upon dechorionation were videotaped (Fig. 1A arrow, Fig. 1B), segregated from the motile embryos and raised until 48 hpf. Non-motile embryos did not respond to tactile stimulation applied to their tails at 30 hpf. This behavioral response is mediated by RB neurons (Ribera & Nusslein-Volhard, 1998). Immunohistochemistry was performed on these embryos using aat and zn12 to confirm the presence or absence of RB neurons, as *nrd*^−/−^ embryos lack RB neurons (Artinger *et al.*, 1999). Anatomical analysis confirmed that the embryos identified as non-motile at 21–22 hpf (Fig. 1B), lacking tail evoked touch responses at 30 hpf, did not have RB neurons, indicating that they were *nrd*^−/−^ embryos (*n*= 37; Fig. 1C and D). The *nrd* mutants also have alterations in pigmentation (Artinger *et al.*, 1999) and this phenotype was seen in non-motile embryos. However, we felt it was initially important to directly confirm the presence or absence of RB neurons when determining the identity of non-motile embryos, and thus focused on the RB cells. The embryos that were motile at 21–22 hpf (*n*= 146) exhibited tail evoked touch responses and had RB neurons.

### *nrd* mutants fail to exhibit spontaneously occurring movements while in the chorion

In developing zebrafish, spontaneous muscle contractions occur between 18 and 27 hpf and are driven by nervous system activity (Melancon *et al.*, 1997; Saint-Amant & Drapeau, 1998; Downes & Granato, 2006). These early contractions are the result of primary motoneurons releasing acetylcholine and exciting post-synaptic muscle pioneers and fibers adjacent to the muscle pioneers (Melancon *et al.*, 1997).

**Fig. 2:**
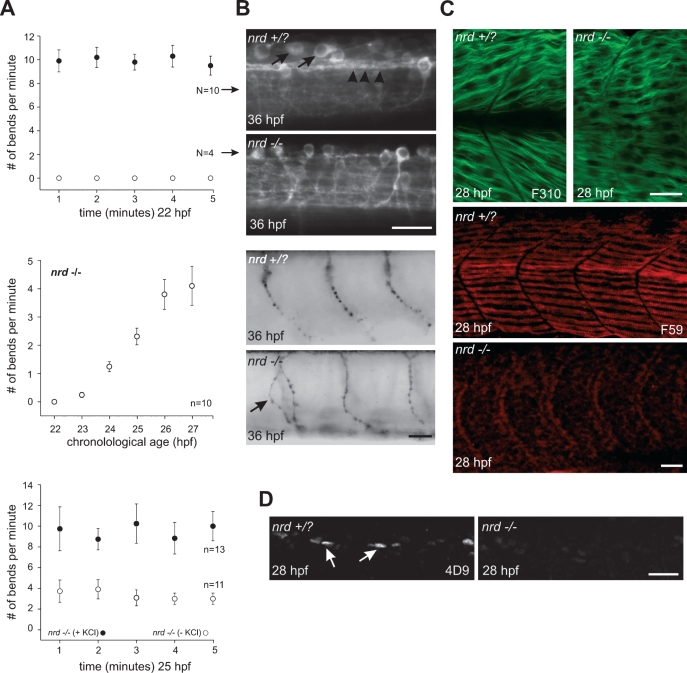
Behavioral characterization, motoneuron and muscle anatomy in *nrd*^−/−^ embryos. (A) Top, while still in their chorions, embryos were separated based on lack of movement. At 36 hpf, they were fixed and processed for aat immunohistochemistry. Middle, prior to 22 hpf, *nrd*^−/−^ embryos do not exhibit spontaneous twitches of the musculature. Ultimately, *nrd*^−/−^ embryos exhibit spontaneous twitches of the musculature at ∼24 hpf. Bottom, *nrd*^−/−^ embryos raised in embryo medium containing high [K^+^] (filled circles) exhibited a significant increase in bend rate compared with *nrd*^−/−^ embryos raised in normal embryo medium (open circles). (B) Top, the embryos that exhibited movement possessed RB neurons as shown by black arrows pointing to RB somata and black arrowheads pointing to the dorsal longitudinal fasciculus (DLF). The DLF partially comprises axons of RB neurons. Thus, these embryos were *nrd*^*+/?*^ siblings. Those that did not move lacked RB neurons and were *nrd*^−/−^ embryos. In the grey (inverted) panels, we also observed that the ventrally projecting motoneuron axons in embryos that lacked RB neurons (*nrd*^−/−^) had abnormal branching patterns (black arrow) as shown in the very bottom panel. (C) Top, photomicrographs of 28-hpf *nrd*^*+/?*^ and *nrd*^−/−^ embryos labeled with the antibody F310, which detects fast muscle fibers. Bottom, photomicrographs of 28-hpf *nrd*^+/?^ and *nrd*^−/−^ embryos labeled with the antibody F59, which detects slow muscle fibers. (D) Photomicrographs of 28 hpf *nrd*^*+/?*^ and *nrd*^−/−^ embryos labeled with the antibody 4D9, which detects *engrailed* expressing muscle pioneers, show lack of engrailed expression in *nrd*^−/−^ embryos. Scale bars, 20 μm.

The fact that *nrd*^−/−^ embryos were non-motile upon dechorionation suggested to us that they could lack embryonic motor activity while still in the chorion. *nrd*^−/−^ embryos were behaviorally and anatomically examined to determine if this was true. In these experiments, 14–17 embryos (19–23 hpf) from a clutch spawned from parents heterozygous for the *narrowminded* mutation were placed in a Petri dish and observed for a minimum of 5 min. When observed prior to 22–23 hpf, a fraction of these embryos still in the chorion were non-motile. The embryos were then videotaped in 5-min epochs every hour until 27 hpf keeping track of the identified non-motile embryos. After this, the non-motile embryos were transferred to separate Petri dishes and raised until 36–48 hpf. This type of experiment was performed three times (*n*= 47 embryos). In all, the 13 embryos that we predicted to be *nrd*^−/−^ based on behavior analysis in this manner (Fig. 2A, top) turned out to be *nrd*^−/−^ embryos upon anatomical analysis as they lacked RB neurons (Fig. 2B, top, monoclonal antibody aat). The remaining 34 that were predicted to be *nrd*^*+/?*^ were indeed *nrd* siblings possessing RB neurons (Fig. 2B, top).

We wanted to be able to identify *nrd*^−/−^ embryos early during embryogenesis and while still in their chorions for behavioral experiments. Once confident that we could segregate and identify *nrd*^−/−^ embryos from their siblings solely on the lack of spontaneously occurring muscle contractions while still in their chorions (Fig. 2A, top), we screened dishes of embryos, pulling out the non-motile ones predicting that they would be *nrd*^−/−^. In total, all 78 non-motile embryos screened in this way were confirmed to be *nrd*^−/−^ embryos lacking RB neurons.

Wild-type and *nrd*^*+/?*^ embryos begin to exhibit contractions of the musculature as early as 18–19 hpf, whereas *nrd*^−/−^ embryos begin having twicthes of the musculature at 24–25 hpf. Thus, the onset of spontaneously occurring activity, as revealed by trunk musculature contractions, is delayed by approximately 6 h in *nrd*^−/−^ embryos (Fig. 2A, middle). Moreover, even when motor activity appeared in *nrd*^−/−^*embryos*, the level of activity observed in 24–25 hpf *nrd* mutants (∼2 bends per minute) was still lower than that seen for 24–25 hpf wild-type embryos in their chorions (Thomas *et al.*, in press).

### *nrd* mutants move when exposed to KCl

*nrd*^−/−^ embryos were segregated from their siblings at 19–21 hpf based on their lack of movement. At this point, some of these mutants were transferred into embryo medium containing 125 mm KCl (high [K^+^]) and some were kept in fresh embryo medium (control). At 25 hpf, the behaviors of both control and high [K^+^]-exposed embryos were videotaped and subsequently analyzed. *nrd*^−/−^ embryos reared in embryo medium containing high [K^+^] had a higher rate of musculature twitches than *nrd*^−/−^ embryos reared in normal embryo medium (Fig. 2A, bottom), suggesting that the nervous system and/or muscle were depolarized and were more active than in the *nrd*^−/−^ embryos not raised in high [K^+^] embryo medium.

The anatomical analysis described earlier confirmed that the non-motile embryos early in development were *nrd*^−/−^ embryos lacking RB neurons. This was done via immunohistochemistry where the monoclonal antibody aat, a marker which reliably labels primary neurons such as RB neurons and motoneurons (Svoboda *et al.*, 2001), was used to reveal the lack of RB neurons in candidate *nrd*^−/−^ embryos. In these initial experiments, aat labeling in *nrd*^−/−^ embryos also showed that motoneurons had problems in pathfinding and exhibited ectopic branches (Fig. 2B, bottom).

The mutated gene in *narrowminded* is the transcription factor *prdm1* (Hernandez-Lagunas *et al.*, 2005) and this allele is also mutated in *u-boot* (*ubo*) mutants (Roy & Ng, 2004). Along with its involvement in neural crest and RB neuron development, *prdm1* is involved in the final step of slow muscle development, which is the differentiation of adaxial cells into laterally located slow muscle fibers and medially located muscle pioneers. In *ubo* mutants, those adaxial cells destined to become mononucleated slow muscle instead become multinucleated fast muscle fibers (Roy *et al.*, 2001; Baxendale *et al.*, 2004). The fast muscle in 28 hpf *nrd*^−/−^ embryos appeared normal when analyzed with immunohistochemistry (Fig. 2C, top). Consistent with *ubo* mutant muscle anatomy, slow muscle fibers were not detected in young *nrd*^−/−^ embryos (28 hpf) with F59 labeling (Fig. 2C, bottom). Moreover, the mutants lacked muscle pioneers as revealed by staining with the antibody 4D9 (Fig. 2D).

### Primary motoneuron axonal pathfinding errors in *nrd*^−/−^ embryos

The experiments using aat revealed a problem in motoneuron axon development (Fig. 2B). Because *nrd*^−/−^ embryos lack adaxial cells it was possible that this was the cause of the altered phenotype. However, the phenotypes of altered axonal pathfinding and ectopic branching were different from the motoneuron phenotypes observed in zebrafish *you-too* (*yot*) mutants, which also lack adaxial cells. In *yot* mutants, many of the primary motoneuron axons never appear to exit the spinal cord (van Eeden *et al.*, 1996). This likely contributes to the severe motility deficit observed in *yot* mutants throughout development. Thus, we performed a more rigorous analysis of motoneuron morphology via immunohistochemistry using antibodies that label either primary motoneuron axons (znp1) or secondary motoneurons and their corresponding axons (zn5), predicting that the axonal phenotypes would differ from those observed in *yot* mutants.

**Fig. 3:**
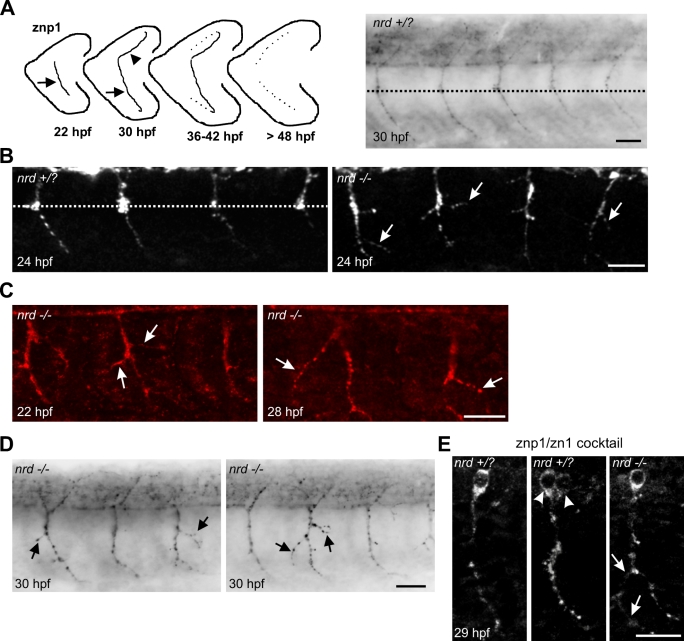
Motoneuron pathfinding errors in *nrd*^−/−^ embryos. The antibody znp1 was used to label primary motoneuron axons in *nrd*^*+/?*^ and *nrd*^−/−^ embryos. (A) Left, cartoon depicting the pattern of znp1 labeling observed in 22- to 48-hpf embryos. Initially, the ventral axon (CaP, arrows) projects ventrally and then turns back to the midline. The dorsal projecting axon (MiP, arrowhead) follows a similar trajectory to the dorsal periphery and then turns and projects to the midline. The dotted lines represent axonal trajectories to the midline that are in a different focal plane compared with the focal plane of the axons projecting to the distal periphery (solid lines). Right, znp1 labeling in a 30-hpf embryo showing the characteristic trajectories of primary motoneuron axons. The dotted line corresponds to the region of the choice point. When CaP axons make it to the choice point, they turn caudally and project into the periphery. (B) Photomicrographs of a 24-hpf *nrd*^*+/?*^ embryo (left) and a 24-hpf *nrd*^−/−^ embryo (right). Note that the axons in the *nrd*^−/−^ embryo do not exhibit the change in trajectory at the choice point like those axons in the *nrd*^*+/?*^ embryo. There are also ectopic branches projecting off these axons (arrows) in the mutant. (C) Photomicrographs of two *nrd*^−/−^ embryos, 22 hpf and 28 hpf. There are ectopic branches projecting off the CaP axons (arrows). (D) Photomicrographs of two 30 hpf *nrd*^−/−^ embryos. There are ectopic branches projecting off the ventral axons (arrows). (E) Photomicrographs of ventral motoneuron axons in *nrd*^*+/?*^ and *nrd*^−/−^ embryos*.* The motoneurons were revealed using znp1/zn1 immunohistochemistry. Using this procedure, somas of CaP and VaP (white arrowheads) were easy to detect as well as the ventral projecting axon. In the *nrd*^−/−^ embryo (right), the axon shown has an ectopic branch (white arrow), but only one motoneuron soma is present. It is likely a CaP motoneuron. In this example, the ectopic branching is not from the VaP motoneuron living longer in this segment. Scale bars, 20 μm.

In zebrafish, the primary motoneurons are born during gastrulation, whereas secondary motoneurons are born later (Beattie *et al.*, 1997). Beginning at ∼22–24 hpf, primary motoneurons can be identified based on their axonal trajectories as well as their cell body positions within each hemi-segment (Eisen *et al.*, 1986; for a review, see Lewis & Eisen, 2003). MiP (middle primary) motoneurons have axons that project dorsally whereas CaP (caudal primary) motoneuron axons innervate the ventral trunk muscle. Another primary motoneuron known as VaP (variable primary) typically undergoes apoptosis by 36 hpf (Eisen *et al.*, 1990). The axons of VaP motoneurons innervate the ventral region of dorsal musculature and they do not extend beyond the horizontal myoseptum. Between 40 and 72 hpf, presumed MiP axons have reached their furthest destination in the dorsal periphery and loop back to the horizontal myoseptum of the embryo. Along their path, they extend from the medial part of the muscle into the more lateral aspects of the periphery. The ventral axons of presumed CaP motoneurons follow a similar path, reaching their most distal destination in the ventral periphery and then loop back to the horizontal myoseptum of the embryo (Fig. 3A). Even in embryos that have had their muscle pioneers ablated, the CaP axon still targets to the periphery in a normal fashion (Melancon *et al.*, 1997). The right panel in Fig. 3A depicts CaP and MiP axonal trajectories in a 30 hpf *nrd*^*+/?*^ embryo labeled with the monoclonal antibody znp1. This pattern is very similar to that observed in stage-matched wild-type embryos (data not shown). We then utilized znp1 to label primary motoneuron axons in *nrd*^−/−^ embryos to determine if they were abnormal when compared with their stage-matched siblings or wild-type embryos.

The axons of CaP motoneurons in *nrd*^−/−^ embryos exhibited detectable abnormalities as early as 22 hpf. These axons often had extra branches and took abnormal trajectories into the periphery (Fig. 3B–E). Not all of the CaP axons were affected, but this phenotype was nonetheless very robust in *nrd*^−/−^ embryos. After 28 hpf, it may not necessarily be appropriate to call the ventral projecting motoneuron axons CaP axons because interpretations in the literature suggest that znp1 only can be used to detect CaP axons prior to 28 hpf. Nonetheless, these ventrally projecting motoneuron axons were still altered.

The ectopic branching could be related to the VaP motoneuron living longer than normal in the *nrd*^−/−^ embryos. VaP motoneuron cell death is governed by the presence of muscle pioneers. In embryos lacking muscle pioneers, the VaP motoneuron lives and its axon projects ventrally into the periphery. This is often associated with ectopic branching off the CaP axon (Eisen & Melancon, 2001). To determine if the ectopic branching associated with the *nrd*^−/−^ ventral motoneuron axons was related to VaP motoneurons living longer than they normally should, we used a znp1/zn1 cocktail to label both VaP and CaP motoneuron cell bodies and the ventral axon associated with these motoneurons (Fig. 3E) (Hutchinson & Eisen, 2006; Hutchinson *et al.*, 2007). In *nrd*^*+/?*^ embryos, a single ventral axon was always associated with either CaP motoneuron somata alone or with the CaP/VaP soma doublet (Fig. 3E, left and middle panels). When we detected ectopic branching in the mutants, it was invariably associated with a single soma, presumably the CaP motoneuron (Fig. 3E, right).

### Secondary motoneuron axonal pathfinding errors in *nrd*^−/−^ embryos and larvae

**Fig. 4:**
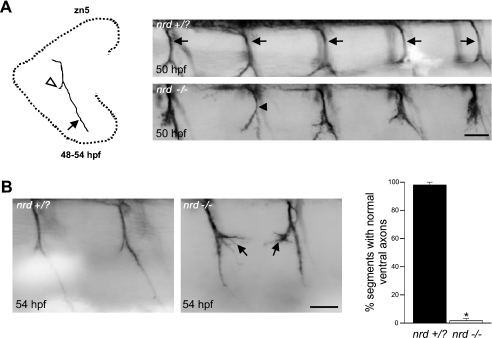
*nrd*^−/−^ embryos and secondary motoneuron axon abnormalities. The antibody zn5 was used to label secondary motoneuron axons in *nrd*^*+/?*^ and *nrd*^−/−^ embryos. (A) Left, cartoon depicting the pattern of zn5 labeling observed in 48–54-hpf embryos. At this stage of development, a prominent ventral–medial projection is apparent (arrow) but no dorsal projection is detected by zn5. A ventral–lateral projection is also present (open arrowhead). Right, photomicrographs of ∼50-hpf *nrd*^*+/?*^ and *nrd*^−/−^ embryos processed for zn5 immunohistochemistry. In the *nrd*^*+/?*^ embryo, arrows point to ventral axons that exit the spinal cord in a tight fascicle. In the *nrd*^−/−^ embryo, four of the five segments shown have axons that appear to be unraveling. The ventral projection labeled by the arrowhead appears normal. However, it appeared to have prematurely forked into two branches compared with those axons in the *nrd*^*+/?*^ embryo. (B) Photomicrographs of 54-hpf *nrd*^*+/?*^ and *nrd*^−/−^ embryos processed for zn5 immunohistochemistry. Arrows in the *nrd*^−/−^ embryo emphasize axons that exhibit pathfinding problems as extra branches are apparent. To the right, quantification indicated that almost all of the segments analyzed in *nrd*^*+/?*^ embryos had normal ventral axons (98 ± 2.0%, *n*= 52). In contrast, only 2 ± 1.7% of the segments analyzed (*n*= 61) in *nrd*^−/−^ embryos had normal ventral axons. Scale bar, 20 μm. *P< 0.05.

**Fig. 5:**
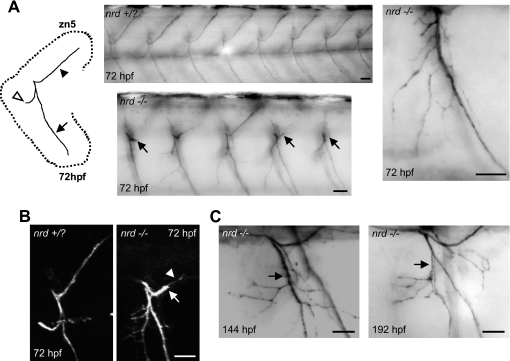
*nrd*^−/−^ larval zebrafish have abnormal secondary motoneuron axon morphology. The antibody zn5 was used to label secondary motoneuron axons in *nrd*^*+/?*^ and *nrd*^−/−^ larvae. (A) Left, cartoon depicting the pattern of zn5 labeling observed in 72-hpf larvae. At this stage of development, a prominent ventral–lateral projecting nerve projection is present (open arrowhead) along with the ventral medial nerve (arrow) and dorsal nerve (arrowhead). Middle, zn5 labeling in a 72-hpf *nrd*^*+/?*^ larva and a 72-hpf *nrd*^−/−^ larva. Arrows point to dorsal axons which appear to be stunted and do not project to the periphery. The ventral axons also appear abnormal when compared with the control. To the right is a high-magnification photomicrograph of a ventral projecting secondary motoneuron nerve from a 72-hpf *nrd*^−/−^ larva. Note the extra branching. (B) Three-dimensional projections from the trunk regions of 72-hpf *nrd*^*+/?*^ and *nrd*^−/−^ larvae. As was the case for the single focal plane image shown in A, the dorsal projecting nerve in the *nrd*^−/−^ larva appeared to have stalled as it targeted to the periphery (white arrow). The white arrowhead points to a thin portion of the nerve where it appears that the axons resumed targeting to the periphery after being stalled. The ventral projection also exhibits ectopic branching. (C) High-magnification photomicrographs of ventral projecting secondary motoneuron axons from 144-hpf and 192-hpf *nrd*^−/−^ larval zebrafish. Black arrows point to ventral projecting motoneuron axons and not the processes associated with dorsal root ganglion neurons. Scale bars, 20 μm.

The secondary motoneurons are the population of motoneurons that comprise most of the motor pool in zebrafish and innervate both fast and slow muscle fibers. They are born in later rounds of neurogenesis after the primary motoneurons. They begin extending their axons at 23 hpf and extend into the periphery by following the axonal pathways that have been laid down by the primary motoneurons (for a review, see Lewis & Eisen, 2003). Secondary motoneurons were analyzed in *nrd*^−/−^ embryos with the antibody zn5 to determine if their axonal trajectories had problems in pathfinding similar to the CaP axons of *nrd*^−/−^ embryos. At 48 hpf, zn5 reliably labels secondary motoneuron somata and their ventral projecting axons (Ott *et al.*, 2001; Fig. 4). When secondary motoneurons in *nrd*^−/−^ embryos were labeled with zn5, the ventral nerve projections had problems in pathfinding by 48–50 hpf when compared with stage-matched siblings or wild-type embryos (Fig. 4A and B). In contrast to secondary motoneuron axons of *yot* mutants (54 hpf; Zeller *et al.*, 2002), which largely fail to exit the spinal cord and enter the periphery, secondary motoneuron axons in *nrd*^−/−^ embryos exited the spinal cord in a normal manner. However, after exiting the spinal cord, the axons in the nerve bundle became unraveled (Fig. 4A). By 72 hpf and continuing through 192 hpf, the ventral secondary motoneuron axons also had numerous ectopic branches (Fig. 5A–C). Also, in many instances, the dorsal projecting axons innervating the dorsal myotomes had not completely extended into the periphery by 72 hpf (Fig. 5A, middle, and Fig. 5B, right).

### Muscle physiology and morphology in 72 hpf *nrd*^−/−^ larval zebrafish

*nrd*^−/−^ larvae are capable of spontaneously swimming after 72 hpf, which indicates that the motoneurons and muscle are clearly active at this developmental stage. The fact that the ventral projections remained hyper-branched until 192 hpf was perplexing as the motoneurons and muscle were now clearly active. We were concerned that the fast muscle development in the *nrd*^−/−^ embryos could be altered during development. If the fast muscle was compromised in *nrd*^−/−^ embryos, this could alter motoneuron development at later developmental stages. Also, as fast muscle physiology and morphology at developmental stages later than 24 hpf have not been characterized for the two mutants lacking *prdm1* (*narrowminded* and *u-boot*), we thought it was necessary to perform an analysis of muscle morphology and physiology at later developmental stages in *nrd*^−/−^ larvae.

Moreover, it has been shown in zebrafish that overactivity of muscle fibers affects primary motoneuron axonal pathfinding. In the *twister* mutant (*nic1*^*twister*^), a mutation in the muscle-specific nicotinic acetylcholine receptor (nAChR) subunit known as chrna1 prolongs the open time of the muscle-specific nAChR when bound to acetylcholine. Thus, during early development, the receptor is overactive and causes muscle degeneration in embryos homozygous for the *twister* mutation (Lefebvre *et al.*, 2004). Even in heterozygous *twister* mutants, the chrna1 containing nAChR is overactive. However, the muscle anatomy appears relatively normal in those heterozygote embryos. Concurrently, primary motoneuron axons in *twister* mutants exhibit ectopic branches. This branching is more severe in the homozygous *twister* mutants vs. heterozygote *twister* siblings.

**Fig. 6:**
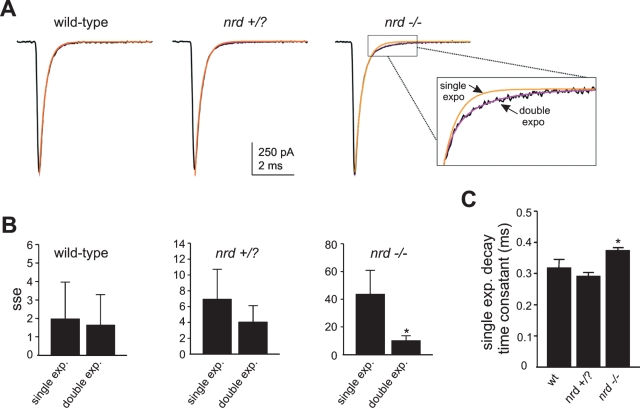
Muscle physiology in *nrd*^*+/?*^ and *nrd*^−/−^ larval zebrafish. (A) Averaged traces of 26–48 mEPCs shown with single (orange) and double (magenta) exponential fits for the decay component obtained from 72-hpf larval zebrafish. (B) Bar graphs quantifying the SSE for the single and double exponential fits of the decay component for the traces shown in A, wild-type (*n*= 6), *nrd*^*+/?*^ (*n*= 8) and *nrd*^−/−^ (*n*= 17). The SSE for a double exponential fit is significantly smaller for the *nrd*^−/−^ mutants (*P< 0.05, *n*= 17). (C) Bar graphs of single exponential fits for the decay component showing a significantly longer decay for the mEPCs acquired from *nrd*^−/−^ larval zebrafish.

We sought to determine if there were any changes in the properties of mEPCs (muscle synaptic physiology) arising from the activation of nAChRs associated with axial muscles in *nrd*^−/−^ larvae because there was a lack of activity and an increase in the branching pattern of primary motoneurons in these fish. Primary motoneurons are not thought to innervate slow fibers (van Raamsdonk *et al.*, 1983; de Graaf *et al.*, 1990; van Asselt *et al.*, 1993) and the slow fibers were never present in the *nrd*^−/−^ larvae; therefore, we only recorded mEPCs from fast fibers. We found that there was a small but significant change in the decay kinetics of the mEPCs, while the majority of cellular and other mEPC properties were unchanged between wild-type and mutant larvae (Table 2). Specifically, we found that the mEPCs from *nrd*^−/−^ larvae were significantly better fit with a double exponential decay rather than a single exponential decay as in the wild-type and heterozygous siblings (Fig. 6A). A comparison of the SSEs (Fig. 6B) shows that a double exponential fit is significantly better than a single fit for the mEPCs from *nrd*^−/−^ larvae (*P*< 0.05), suggesting that there is a change in the off-kinetics of mEPCs in *nrd*^−/−^ larvae. Furthermore, we found that mEPCs recorded from fast fibers of *nrd*^−/−^ larvae took significantly longer to decay to baseline (τ = 0.37 ± 0.01 ms, *n*= 17) than heterozygous siblings (τ = 0.29 ± 0.01 ms, *n*= 8) and wild-type fish (τ = 0.32 ± 0.02 ms, *n*= 6) when the decay was forced-fit with a single exponential component (Fig. 6C, *P*< 0.05). Even though we detected a change in muscle physiology, where the decay times of mEPCs in the mutants differed from siblings, this difference did not compare with the differences in decay times documented for *twister* mutants and stage-matched *twister* siblings (see Lefebvre *et al.*, 2004; Figure 6). In that study, decay time differences were of the order of 100 ms, whereas in *nrd*^−/−^ larvae, the differences in mEPC decay times was about 0.08 ms (see Table 2 for summary). Thus, we conclude that the muscle-specific nAChR is not overactive in *nrd*^−/−^ larvae and does not likely contribute to the motoneuron pathfinding errors.

**Table 2:** Comparison of electrophysiological parameters obtained from 72-hpf wild-type, *nrd*^*+/?*^ and *nrd*^−/−^ larvae

Parameters	Wild type (*n*= 6)	*nrd*^*+/?*^ (*n*= 8)	*nrd*^−/−^ (*n*= 17)
Amplitude (pA)	1089 ± 111	990 ± 102	838 ± 77
Rise time (ms)	0.065 ± 0.003	0.073 ± 0.003	0.074 ± 0.003
τ (ms)	0.32 ± 0.02	0.29 ± 0.01	0.37 ± 0.01*,†
R_m_ (MΩ)	125 ± 19	65 ± 7*	133 ± 16†
Capacitance (pF)	66 ± 6	72 ± 9	56 ± 3†

* Values significantly different from wild-type fish.

† Values significantly different from *nrd*^*+/?*^.

**Fig. 7:**
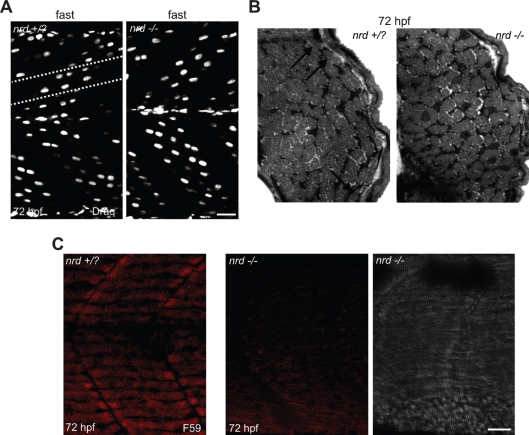
Muscle morphology in *nrd*^*+/?*^ and *nrd*^−/−^ larval zebrafish. (A) Confocal images from 72-hpf *nrd*^*+/?*^ and *nrd*^−/−^ larvae labeled with the vital dye *Draq 5* to reveal nuclei of fast muscle. Nuclei of fast muscle have a characteristic signature as they are ‘tilted’ at 45° (white dashed lines). (B) Cross-sections through muscle of 72-hpf *nrd*^*+/?*^ (left) and 72-hpf *nrd*^−/−^ larval zebrafish (right). Arrows in the *nrd*^*+/?*^ larva point to slow muscle fibers at the lateral aspect of the musculature. These slow fibers were not observed in *nrd*^−/−^ larval zebrafish. (C) Photomicrographs of 72-hpf *nrd*^*+/?*^ (left) and 72-hpf *nrd*^−/−^ larvae (middle) labeled with the antibody F59 to detect slow muscle fibers. The *nrd*^−/−^ larva lacks F59, slow muscle fiber labeling. Right, differential interference contrast image of the 72-hpf *nrd*^−/−^ larva. Scale bars, 20 μm.

We also analyzed features of muscle anatomy in 72 hpf *nrd*^−/−^ larvae to see if they were abnormal. The transcription factor *prdm1* is required for slow muscle differentiation. In *ubo* mutants where *prdm1* is mutated, slow muscle fibers switch their fates and become fast muscle (Baxendale *et al.*, 2004). We analyzed the distribution of muscle nuclei to see if more lateral fast muscle was present in the *nrd*^−/−^ larvae. *Draq 5*, a vital dye used to label cell nuclei in living as well as fixed tissue, reliably labeled fast muscle nuclei in the *nrd* siblings and *nrd*^−/−^ larvae (Fig. 7A) with no obvious differences between the labeling patterns in lateral white muscle fibers where the electrophysiological recordings were obtained.

In cross-sections, fast muscle in *nrd*^−/−^ larvae also appeared normal when compared with their siblings. Normal fast muscle was also seen during our dissections preparing the larvae for electrophysiological investigations. In the cross-sections obtained from 72 hpf larvae, we could not detect slow muscle fibers (Fig. 7B). Consistent with this observation, 72 hpf *nrd*^−/−^ larvae were devoid of F59 labeling, indicating that they lacked slow muscle fibers (Fig. 7C).

### Restoring activity with KCl rescues motoneuron axonal morphology

**Fig. 8:**
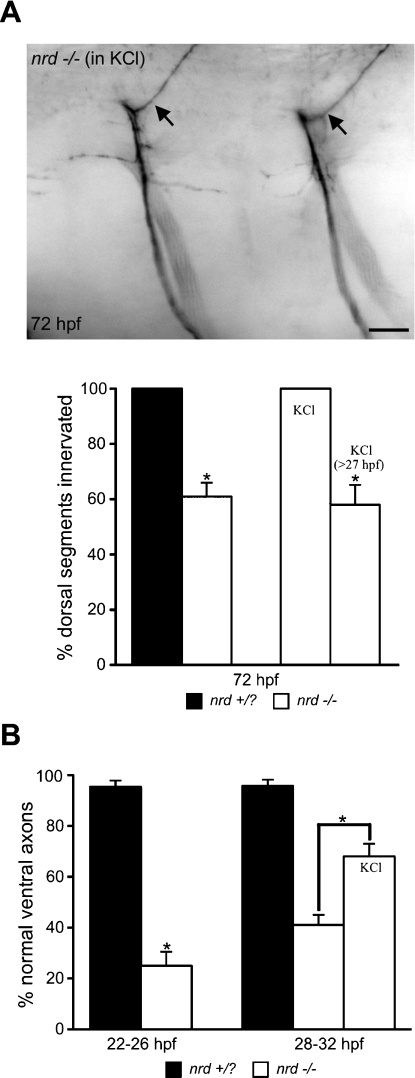
Depolarizing activity early in development restores motoneuron morphology in *nrd*^−/−^ larvae. (A) Morphology of secondary motoneurons was analyzed in larvae exposed to high [K^+^] utilizing the antibody zn5. In 72-hpf, high [K^+^]-exposed *nrd*^−/−^ larvae, the dorsal axons project well into the periphery (compare with Fig. 5). The ventral nerve projections appeared to have less axonal branches than *nrd*^−/−^ larvae not exposed to high [K^+^]. The percentage of dorsal projections that extended into the periphery was quantified to determine the extent of ‘restoration’ back to the normal phenotype caused by raising embryos in embryo medium containing high [K^+^]. In 72-hpf *nrd*^−/−^ larvae, 61 ± 5.0% of dorsal segments (*n*= 100) were innervated by a nerve compared with 100% in wild-type or sibling controls. In the high [K^+^] exposed *nrd*^−/−^ larvae, 100% of the dorsal segments analyzed (*n*= 65) were all innervated. In 72-hpf *nrd*^−/−^ larvae reared in high [K^+^] embryo medium after 27 hpf, 58 ± 7.2% of the dorsal segments analyzed (*n*= 61) were innervated by secondary motoneuron axons. (B) Quantification further revealed that primary motoneuron axonal development was influenced by depolarizing activity. In 22- to 26-hpf *nrd*^−/−^ embryos, 25 ± 5.5% of the CaP axons (*n*= 52) were normal. Abnormalities in the form of ectopic branching were observed in the remaining CaP axons. By 28–32 hpf, 41.0 ± 4.0% of the ventral projecting motoneuron axons were normal (*n*= 76). The remaining ventral axons exhibited ectopic branching or pathfinding errors. In contrast, ∼95% (103/108 total axons) of all the ventral motoneuron axons analyzed in 22- to 32-hpf *nrd*^*+/?*^ embryos were normal. In *nrd*^−/−^ embryos raised in high [K^+^] embryo medium, 68 ± 5.0% of the ventral primary motoneuron axons (*n*= 37) had a normal morphology. The remaining axons were still abnormal, exhibiting ectopic branching. Scale bar, 20 μm. *P< 0.05.

We showed earlier that exposing embryos early in development to KCl provided a depolarizing drive which caused the embryos to move. We hypothesized that this depolarizing drive could potentially restore motoneuron anatomy. In 72 hpf *nrd*^−/−^ larvae reared in high [K^+^] embryo medium beginning at 19–21 hpf, dorsal projecting secondary motoneuron nerves extended well into the dorsal myotome innervating the dorsal segments. Moreover, the main ventral projecting axons as well as the dorsal projecting axons appeared very organized and had very stereotypic trajectories. At the qualitative level, the axons appeared to exhibit less branching (Fig. 8A). In essence, these secondary motoneuron axons resembled axons of stage-matched *nrd*^*+/?*^ larvae or wild-type larvae (refer to Fig. 5A)*.* Conversely, raising *nrd*^−/−^ larvae in high [K^+^] embryo medium after 27 hpf had no effect on motoneuron morphology. The motoneuron axons still exhibited numerous ectopic branches and many of the dorsal axons failed to project to the periphery by 72 hpf (Fig. 8A, bottom).

The antibody zn5 recognizes a cell adhesion molecule known as DM-GRASP (Fashena & Westerfield, 1999). In zebrafish, DM-GRASP is down-regulated in secondary motoneuron somata by 4–5 dpf (Fashena & Westerfield, 1999). In our previous study investigating the effects of nicotine exposure on embryos (Svoboda *et al.*, 2002), we noticed that the down-regulation of zn5 expression coincided with dorsal axons extending into the periphery. In *nrd*^−/−^ larvae raised in embryo medium containing high [K^+^], the somatic expression of DM-GRASP was reduced compared with DM-GRASP expression in *nrd*^−/−^ larvae raised in normal embryo medium (not shown).

The above results indicated that depolarizing activity early in development may help guide dorsal motoneuron axonal targeting into the periphery. Our behavior data and anatomical analysis of primary neuron axons suggested that this might be true. At 22 hpf, *nrd*^−/−^ embryos do not exhibit any motor output (Fig. 2A). In those embryos, ∼25% of the ventral motoneuron axons, we refer to as CaP axons, had normal morphologies. As *nrd*^−/−^ embryos age, they start to move and by 27 hpf they have bend rates of about four bends per minute (Fig. 2A, middle). In these older embryos (28–32 hpf), ∼42% of the ventral axons exhibited normal morphologies (Fig. 8B). This morphological ‘recovery’ appeared to coincide with the increased motor activity. To substantiate this phenomenon further, 21 hpf *nrd*^−/−^ embryos were isolated from their siblings and raised in high [K^+^] embryo medium and analyzed at 32 hpf. Prior to the high [K^+^] exposure, these 21 hpf *nrd*^−/−^ embryos did not show any motor output. In the high [K^+^]-exposed mutants, ∼68% of the ventral axons that we presumed to be CaP axons exhibited normal morphologies (Fig. 8B). Wild-type embryos were also raised in embryo medium containing high [K^+^]. This did not produce axonal pathfinding errors for primary motoneurons analyzed at 32 hpf or secondary motoneurons analyzed at 72 hpf (data not shown). Taken together, these data indicate that providing a depolarizing drive during early development in *nrd*^−/−^ embryos is capable of restoring their abnormal motoneuron phenotypes.

### A developmental window of opportunity for motoneuron axonal pathfinding

The results up to this point demonstrate that depolarizing activity early in development is one of many factors, along with muscle-derived cues, that can influence axonal pathfinding. However, *nrd*^−/−^ embryos also lack RB neurons, and these cells may also be needed early in embryogenesis for proper neuronal development to occur. It is likely that RB neurons communicate with other cells as early as 21 hpf, because embryos can perceive touch at this age (Saint-Amant & Drapeau, 1998) and because RB neurons exhibit robust inward sodium currents that are likely capable of supporting action potentials (Ribera & Nusslein-Volhard, 1998; Pineda *et al.*, 2005). If embryos can perceive touch early in development using RB cells, then it is possible that the RB neurons may be able to aid in the generation of the early embryonic motor output.

The *macho* mutants lack a touch response after 27 hpf (Granato *et al.*, 1996; Ribera & Nusslein-Volhard, 1998) as they do not respond to tactile stimulation (tapping) of the tail. This coincides with the age at which RB neurons start to exhibit abnormalities in sodium current and action potential properties, and it is hypothesized that the loss of this tail tap-mediated touch response is caused by defective RB neurons (Ribera & Nusslein-Volhard, 1998). However, RB neurons in *mao*^−/−^ embryos prior to 27 hpf likely have normal physiology. Moreover, *mao*^−/−^ embryos prior to 27 hpf exhibit bends of the musculature and in contrast to *nrd*^−/−^ embryos, they cannot be easily identified from siblings using behavioral criteria (unpublished observations, K.R.S.). Therefore, we reasoned that the motoneuron axons in *mao*^−/−^ should have normal phenotypes because they exhibit embryonic motor behaviors. Unpublished data (K.R.S.) indicate that secondary motoneuron axons in *mao*^−/−^ larvae target to the periphery in a normal manner.

**Fig. 9:**
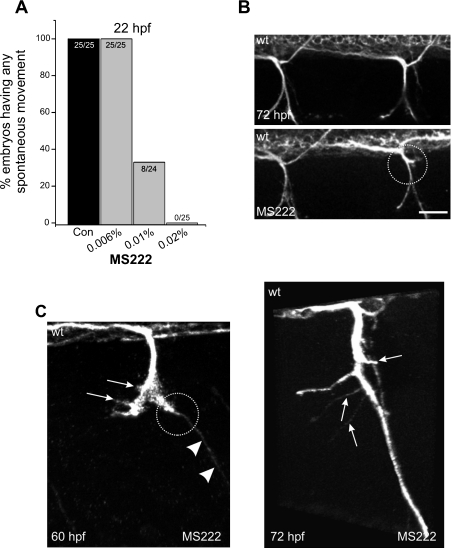
Pharmacological block of neural activity early in development phenocopies aspects of motoneuron morphology seen in *nrd*^−/−^ mutants. (A) Zebrafish embryos were exposed to varying concentrations of MS222 (tricaine) while in their chorions beginning at 15–17 hpf and then returning them to embryo medium at 30 hpf. The embryonic motor output (at 22 hpf) was reduced by 0.01–0.02% tricaine. (B) Photomicrographs of a 72-hpf control larva (top) and a 72-hpf tricaine-exposed larva (bottom). The antibody zn5 was used to label secondary motoneuron axons. The region denoted by the white circle in the bottom panel emphasizes a region where the dorsal projecting nerve appeared to stall, failing to extend into the dorsal myotome. (C) Photomicrographs of two tricaine-exposed zebrafish (60 and 72 hpf, respectively) emphasizing ventrally projecting axons. The antibody zn5 was used to label secondary motoneuron axons. In the 60-hpf embryo at the left, the region denoted by the white circle emphasizes a region where the ventral projecting secondary motoneuron axons appeared to stall before getting to the periphery. They then extended into the periphery appearing as a thin fiber (arrowheads). Ectopic branches (white arrows) are extending off the ventral–lateral projection. In the 72-hpf larva at the right, ectopic branches can be seen denoted by arrows. In these examples, the head is to the left. Scale bar, 20 μm.

Thus, it appeared to us that the depolarizing activity resulting from embryonic motor activity early in development was an important variable in regulating motoneuron development. If this were true, we hypothesized that blocking depolarizing neural activity early in development would alter motoneuron development and alter axonal pathfinding. At 15–17 hpf, embryos were exposed to 0.006–0.02% MS222 (tricaine), an anesthetic that blocks sodium channels. The higher concentration of tricaine abolished the embryonic motor output in embryos at 22 hpf (Fig. 9A). MS222 exposure also affected motoneuron axonal pathfinding. In many instances, the ventral and dorsal projecting secondary motoneuron axons were stalled when exiting the ventral root (Fig. 9B and C). In some instances, the secondary motoneuron axons had ectopic branches, but not to the same extent as seen in the *nrd*^−/−^ larvae (Fig. 9C).

## Discussion

In this study, we characterized the motoneuron anatomy in a mutant zebrafish known as *narrowminded* and investigated the potential role of embryonic motor activity in sculpting spinal neuron development. The study was prompted by the observation that *nrd*^−/−^ embryos have a reduced motor output early in development. We used a combination of behavioral, anatomical and physiological techniques to elucidate the causes of pathfinding errors of motoneuron axons in *narrowminded* mutant embryos.

### Embryonic motility, muscle pioneers and the primary motoneurons

The main behavioral phenotype in *nrd*^−/−^ embryos is the reduced movement that occurs between 18 and 25 hpf. Although muscle pioneers and slow muscle development are altered in *narrowminded*, it is not a paralytic mutant such as *nic1*^*b107*^ (*nic1*) or *sofa potato* (*sop*), both having defects in muscle acetylcholine receptors. In wild-type zebrafish embryos, primary CaP axons are thought to interact with muscle pioneers as early as 17 hpf and this interaction may be responsible for the earliest muscle contractions. In fact, cholinergic activation of muscle pioneers has been shown in embryos as old as 19.5 hpf (Melancon *et al.*, 1997). These contractions, however, may not exclusively be mediated by the medial muscle pioneers. They may also be mediated by muscle fibers immediately adjacent to the muscle pioneers.

We hypothesized that the reduced movement in *nrd*^−/−^ embryos during early embryonic development, even in the absence of the muscle pioneers, may have a neural substrate. We also suspected that motoneurons, during the time when they were projecting to their targets, were inactive. This idea was supported by observations from our behavioral analysis which revealed that the bend rate could be increased by raising embryos in high [K^+^] embryo medium. Thus, the embryos were capable of moving but they just did not move. Moreover, the axonal phenotypes of *nrd*^−/−^ embryos discussed below suggested that the motoneurons were inactive early in embryogenesis.

### Motoneuron axonal pathfinding errors in *nrd* mutants

In zebrafish, several factors contribute to normal axonal pathfinding of primary and secondary motoneurons. These cues are often associated with the periphery such as the muscle pioneers and adaxial cells (Melancon *et al.*, 1997; Zeller *et al.*, 2002). Moreover, exaggerated muscle nAChR activity results in abnormal primary motoneuron development (Lefebvre *et al.*, 2004). Lastly, proper sodium channel expression is also required for normal motoneuron development (Pineda *et al.*, 2006).

In *nrd*^−/−^ embryos, the muscle pioneers are absent and their absence could potentially contribute to the axonal pathfinding deficits. However, it had previously been shown that CaP motoneuron axon morphology appears normal when muscle pioneers are ablated in zebrafish embryos (Melancon *et al.*, 1997). When focusing on what was likely ectopic branching of the CaP axon, only a small fraction of axons exhibited ectopic branches (Melancon *et al.*, 1997). Thus, when the muscle pioneers are ablated in zebrafish, the morphology of CaP axons is not affected to the same extent as the morphology of CaP axons in *nrd*^−/−^ embryos.

We also wondered if the ectopic branching of primary ventral motoneuron axons in *nrd*^−/−^ embryos was due to the VaP motoneurons not entering into a programmed cell death paradigm. As muscle pioneers provide the cue to ‘kill off’ most of the VaP motoneurons, this seemed plausible. However, this possibility was also excluded in the experiments using znp1/zn1 immunohistochemistry. Thus, it appeared that the ectopic branching observed on the ventral projecting CaP axon was not related to the lack of muscle pioneers.

The work of others has clearly established that factors within the extracellular matrix and/or the presence of adaxial cells influences motoneuron axonal pathfinding (Zeller *et al.*, 2002; Schneider & Granato, 2006). In *yot* mutants lacking adaxial cells, both primary and secondary motoneuron axons are severely altered. The *yot* mutants are also immotile and do not appear ever to become motile with time (van Eeden *et al.*, 1996; Zeller *et al.*, 2002).

*nrd* mutants also lack adaxial cells, but the resulting behavioral and anatomical phenotypes are not nearly as severe as those for the *yot* mutants (van Eeden *et al.*, 1996). First, the abnormal motor behavior in *nrd*^−/−^ embryos recovers. Secondly, at the anatomical level, both primary and secondary motoneuron axons extend into the periphery, they just have numerous ectopic branches. This was very obvious for the secondary motoneurons. For example, in 50 hpf *nrd*^−/−^ embryos, ventral secondary motoneuron axons exited the spinal cord, but appeared to unravel quickly (see Fig. 4). One caveat is that the *yot* mutant is associated with altered sonic hedgehog signaling. This may account for the severe abnormal motoneuron phenotypes in those embryos, especially for the secondary motoneuron phenotype (Ingham, 1997). So when assessing the data in the literature, we were puzzled. The motoneuron phenotypes seen in *nrd*^−/−^ embryos lacking adaxial cells were not the same as those motoneuron phenotypes in *yot* mutants also lacking adaxial cells.

The motoneuron phenotypes in *nrd*^−/−^ embryos are, however, reminiscent of neuronal phenotypes from a variety of species that have had their activity blocked either genetically or pharmacologically with tetrodotoxin infusion. However, this phenotype is not consistent with motoneuron axon development in paralytic zebrafish mutants. In both the *nic1* and *sop* paralytic zebrafish mutants, the muscle acetylcholine receptors are non-functional or absent, and hence the muscle is inactive (Westerfield *et al.*, 1990; Ono *et al.*, 2001). The motoneuron axonal morphology in both of these mutants is normal (Westerfield *et al.*, 1990; Welsh *et al.*, in press). Moreover, when muscle pioneers were ablated during zebrafish embryogenesis, the CaP axons still did not exhibit ectopic branching to the extent seen in *nrd*^−/−^ embryos. Thus, if muscle activity is silent, or if muscle pioneers are absent, CaP axonal trajectories are normal. Therefore, we suspected that some other factor had to be causing the ectopic branching and the defasciculation of motoneuron axons. We suspected that this factor was upstream of the muscle and was possibly related to the neural activity of the motoneurons.

### Embryonic motility and RB neurons

In zebrafish, embryonic motor output can occur in spinalized preparations where the hindbrain has been separated from the spinal cord (Downes & Granato, 2006). Motor output can be blocked by strychnine as early as 19 hpf, indicating that these embryonic twitches may be the output of an activated spinal neural circuit (Downes & Granato, 2006). In the spinalized preparation, the mechanosensory RB neuron is in position to drive/excite this embryonic circuit.

*nrd*^−/−^ embryos lack RB neurons and this may also contribute to the reduced embryonic motor output. If an excitatory drive is originating from within the spinal cord as Downes & Granato (2006) have shown, and that excitatory cascade is triggered by RB neurons, the spinal circuit will never be activated and the motoneurons would never be activated. If the function of absent RB neurons is restored by adding back KCl, the excitatory drive may be restored. We hypothesize that KCl may be exciting the interneuronal circuit that the RB neurons make synapses with, thereby providing the excitatory drive that the RB neurons may be providing in the normal embryo. It is also possible that KCl directly depolarizes the motoneurons as well.

We support the idea that the early presence of RB neurons may be providing some type of signaling cue to facilitate activation of the embryonic spinal cord. Because RB neurons are numerous in the embryonic spinal cord, action potential-independent release of glutamate from RB terminals could contribute to the genesis of spontaneous spinal activity. Physiological and behavioral data obtained from *mao*^−/−^ embryos are consistent with these observations. *mao* mutants have abnormally functioning RB neurons, but only after 27 hpf (Ribera & Nusslein-Volhard, 1998). However, the physiology of RB neurons in *mao*^−/−^ embryos resembles the physiology of RB neurons in younger wild-type embryos (Pineda *et al.*, 2005). In accordance with this, *mao*^−/−^ embryos exhibit embryonic twitches of the musculature prior to 27 hpf.

If the reduced twitch rate was due to factors upstream of muscle, this could result in abnormal motoneuron axonal morphologies. If that excitatory drive was restored, as it was when the *nrd*^−/−^ embryos were raised in embryo medium containing high [K^+^], the motoneurons would target to the periphery in an appropriate manner. They would also exhibit much less ectopic branching. The depolarization of muscle alone by KCl would not likely restore the axonal phenotype in light of what we know about the *twi* mutants, in which overactive muscle actually causes ectopic branching of motoneuron axons (Lefebvre *et al.*, 2004).

When *nrd*^−/−^ embryos were raised in embryo medium containing high [K^+^], the embryonic motor output was increased and this coincided with a restored motoneuron axon anatomy. This is an important observation. The muscle did not go into tetanus and seize. Instead, the embryo’s muscle did twitch from side to side, suggesting that the nervous system was activated to produce the output. This is supported by observations from wild-type embryos exposed to high [K^+^]. Under these conditions, KCl activates a motor output where the bends of the musculature alternate. As the muscle in *nrd*^−/−^ embryos did not seize, we suggest that KCl is activating the nervous system upstream of motoneurons, which activates the motor output (see Supporting Information, Fig. S1). We used the behavioral endpoint as a diagnostic tool to demonstrate that the KCl was depolarizing central nervous system elements. The muscle bends were likely due to motoneurons firing action potentials and releasing acetylcholine onto the post-synaptic muscle fibers. At 25 hpf, there was a 2.5-fold increase in motor output in *nrd*^−/−^ embryos raised in high [K^+^] compared with stage-matched mutants not raised in high [K^+^].

### *Nrd*^−/−^ muscle physiology and anatomy

Our muscle physiology and anatomy data also rule against potential muscle defects as a cause of motoneuronal axonal pathfinding errors in *nrd*^−/−^ embryos. In contrast to mEPCs of *twister* mutants, mEPCs in *nrd*^−/−^ embryos are normal. However, a detailed analysis of the mEPCs from fast muscle fibers of *nrd*^−/−^ and wild-type larvae revealed that the decay component is better fit with two exponents in the *nrd*^−/−^ larvae. Single exponential fits have widely been found in a number of adult preparations, but during development, double exponential fits to the decay component of mEPCs have been noted in a number of preparations (Sakmann & Brenner, 1978; Colquhoun & Sakmann, 1981; Brehm *et al.*, 1982). Two exponential fits are largely thought to arise from two different populations of AChRs (embryonic and adult), but may also occur from a change in the kinetics of the open states of a single population of receptors. The gating kinetics of nAChRs can be altered by protein kinase-induced phosphorylation. For instance, phosphorylation of the *Torpedo* nAChR by protein kinase C (Downing & Role, 1987) regulates desensitization rates, while protein kinase A phosphorylation of *Xenopus* muscle nicotinic AChRs mediates recovery from desensitization (Paradiso & Brehm, 1998). The time course of these changes is beyond that observed in the present study, occurring over seconds and minutes rather than on a millisecond timescale. Although these changes in muscle physiology are of interest to us, they probably do not contribute to motoneuron axon pathfinding errors observed in *nrd*^−/−^ embryos and larvae.

### Neural activity as an initiator of axonal targeting in zebrafish

Analysis of the *nrd*^−/−^ embryos coupled with our pharmacological manipulations with both tricaine exposure in wild-type embryos and high [K^+^] exposure established that depolarizing neuronal activity helps target axons to the periphery. Embryonic motor output may be the signature of this activity. As the axons move into the periphery, other factors may contribute to the branching patterns of motoneuron axons. These factors are likely to be linked to adaxial cells which are migrating to the periphery.

Thus, we favor a model where activity initiates the process of axonal targeting. In *nrd*^−/−^ larvae, the axons of secondary motoneurons had two distinct phenotypes. First, ventrally projecting secondary motoneurons had numerous ectopic branches. Ectopic branching of secondary motoneuron axons was also seen in our tricaine studies as well as when Nav_1.6_ was reduced in developing embryos (Pineda *et al.*, 2006). Secondly, in *nrd*^−/−^ larvae as well as tricaine-exposed wild-type embryos, secondary motoneuron axons stalled before entering the periphery. This was another predominant phenotype that was detected when Nav_1.6_ was reduced in zebrafish embryos with morpholino antisense technology (Pineda *et al.*, 2006). Consequently, if sodium channel activity was reduced with molecular or pharmacological manipulations, motor neuron axons were stalled upon entering the periphery.

The final piece to our model is derived from the data obtained from *nrd*^−/−^ embryos raised in high [K^+^] embryo medium. In those larvae, motoneuron axonal phenotypes were restored simply by providing a depolarizing drive during early embryogenesis. Taken together, these findings implicate neural activity in a defined developmental window in helping target motoneuron axons to their peripheral destinations in developing zebrafish.

## Abbreviations

aat: anti-acetylated tubulin
CaP: caudal primary
GFP: green fluorescent protein
hpf: hours post fertilization
LGN: lateral geniculate nucleus
mEPC: miniature endplate current
MiP: middle primary
nAChR: nicotinic acetylcholine receptor
PBST: PBS containing 0.1% Tween 20
RB: Rohon–Beard
SSE: sum of squared errors
VaP: variable primary

## Supporting information

Fig. S1. High [K^+^] embryo medium and its effect on motor output in wild-type zebrafish embryos.
