# Supplementary material for: Embryonic motor activity and implications for regulating motoneuron axonal pathfinding in zebrafish
Source: Eur J Neurosci. 2008 Sep;28(6):1080–96. doi: 10.1111/j.1460-9568.2008.06418.x (PMC2741004; doi:10.1111/j.1460-9568.2008.06418.x)
Supplement: Supplementary file 1 [file ejn0028-1080-SD1.doc]

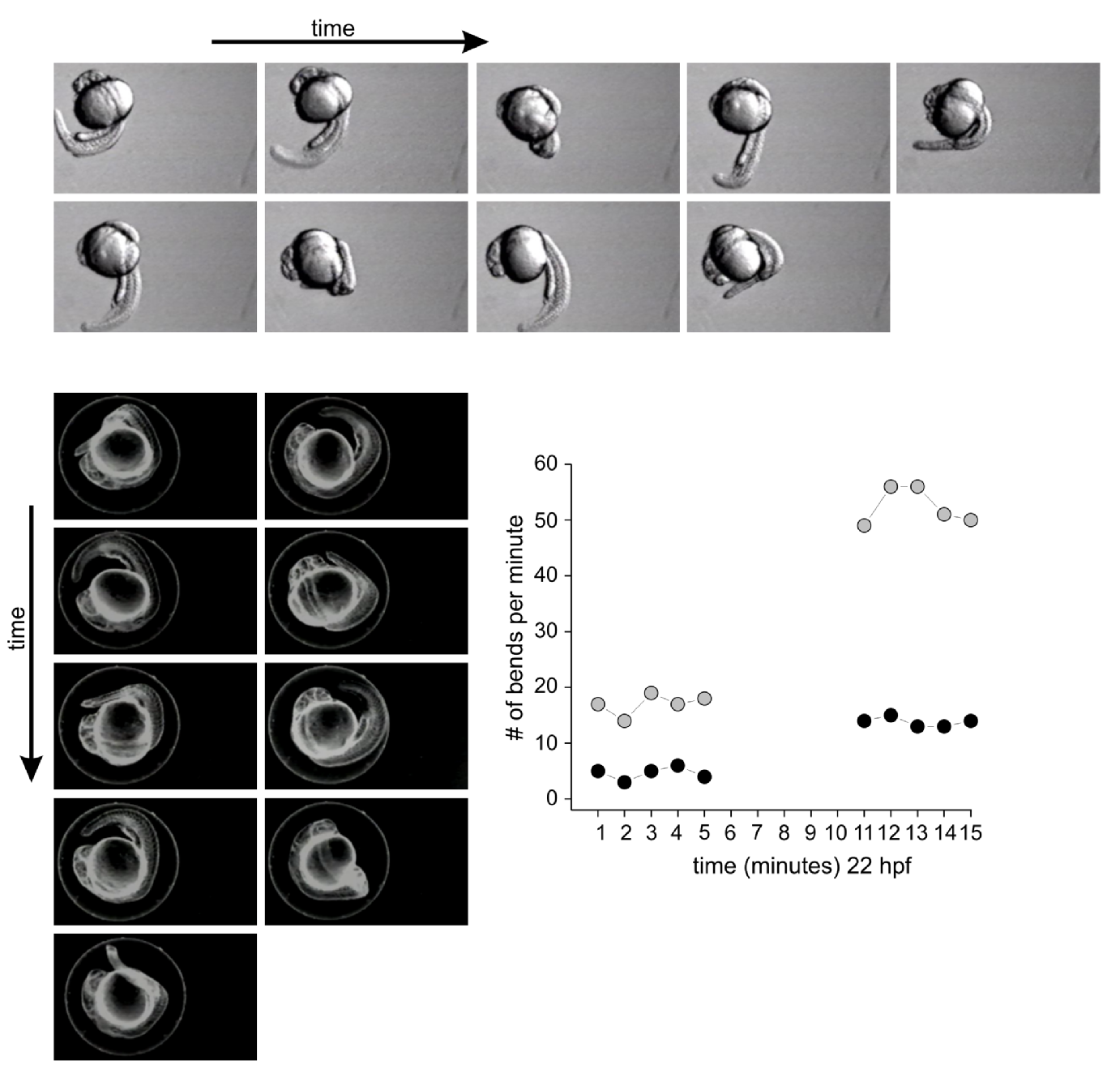


**Fig. S1. High [K^+^] embryo medium and its effect on motor output in wild-type zebrafish embryos.** 22 hpf embryos were placed into embryo medium containing high [K^+^]. A dechorionated embryo shown at the top exhibited a robust motor output with body flexions occurring in a left-right-left fashion. In one stretch, 74 consecutive bends alternated left to right and back to the left again. At the left, an embryo in its chorion was exposed to embryo medium containing high [K^+^]. The embryo moved just like the dechorionated embryo with alternating bends of the musculature. At the right, the behaviors were quantified. In both cases, exposing embryos to high [K^+^] resulted in increased motor output. The ability of KCl to depolarize the CNS and turn on a rhythmic motor output indicates that the neuronal circuit governing early embryonic motility was activated in the high [K^+^] paradigm. Images in grey correspond to dechorionated embryos (grey circles) and images in black correspond to embryos in the chorion (filled circles).
